# Merge Fuzzy Visual Servoing and GPS-Based Planning to Obtain a Proper Navigation Behavior for a Small Crop-Inspection Robot

**DOI:** 10.3390/s16030276

**Published:** 2016-02-24

**Authors:** José M. Bengochea-Guevara, Jesus Conesa-Muñoz, Dionisio Andújar, Angela Ribeiro

**Affiliations:** Center for Automation and Robotics, CSIC-UPM, Arganda del Rey, Madrid 28500, Spain; jose.bengochea@csic.es (J.M.B.-G.); jesus.conesa@csic.es (J.C.-M.); dionisioandujar@hotmail.com (D.A.)

**Keywords:** generation of autonomous behaviour, crop inspection, visual servoing, fuzzy control, precision agriculture, GPS

## Abstract

The concept of precision agriculture, which proposes farming management adapted to crop variability, has emerged in recent years. To effectively implement precision agriculture, data must be gathered from the field in an automated manner at minimal cost. In this study, a small autonomous field inspection vehicle was developed to minimise the impact of the scouting on the crop and soil compaction. The proposed approach integrates a camera with a GPS receiver to obtain a set of basic behaviours required of an autonomous mobile robot to inspect a crop field with full coverage. A path planner considered the field contour and the crop type to determine the best inspection route. An image-processing method capable of extracting the central crop row under uncontrolled lighting conditions in real time from images acquired with a reflex camera positioned on the front of the robot was developed. Two fuzzy controllers were also designed and developed to achieve vision-guided navigation. A method for detecting the end of a crop row using camera-acquired images was developed. In addition, manoeuvres necessary for the robot to change rows were established. These manoeuvres enabled the robot to autonomously cover the entire crop by following a previously established plan and without stepping on the crop row, which is an essential behaviour for covering crops such as maize without damaging them.

## 1. Introduction

Farming practices have traditionally focused on uniform management of the field and ignored spatial and temporal crop variability. This approach has two main negative outcomes: a) air and soil pollution, with consequent pollution of groundwater, and b) increased production costs [1]. Moreover, agricultural production must double in the next 25 years to sustain the increasing global population while utilising less soil and water. In this context, technology will become an essential aspect of minimising production costs while crops and environment are properly managed [2,3,4].

The development of technologies such as global positioning systems (GPS), crop sensors, humidity or soil fertility sensors, multispectral sensors, remote sensing, geographic information systems (GIS) and decision support systems (DSS) have led to the emergence of the concept of precision agriculture (PA), which proposes the adaptation of farming management to crop variability. Particularly important within PA are techniques aimed at selective treatment of weeds (site-specific management) by restricting herbicide use to infested crop areas and even varying the amount of treatment applied according to the density and/or type of weeds, in contrast to traditional weed control methods.

Selective herbicide application requires estimations of the herbicide needed for each crop unit [5]. First, data must be acquired in the field to determine the location and estimated density of the weeds (perception stage). Using this information, the optimal action for the crop is selected (decision-making stage). Finally, the field operations corresponding to the decision made in the previous stage must be performed to achieve the selective treatment of weeds (action stage). At the ground level, data collection can be accomplished by sampling on foot or using mobile platforms. Sampling on foot is highly time-consuming and requires many skilled workers to cover large treatment areas. Discrete data are collected from pre-defined points throughout an area using sampling grids, and interpolation is employed to estimate the densities of the intermediary areas [6].

In continuous sampling, data are collected over the entire sample area. Continuous data enable a qualitative description of abundance (e.g., presence or absence; zero, low, medium, or high) rather than the quantitative plant counts that are usually generated by discrete sampling [7].

To effectively implement PA, the perception stage should be substantially automated to minimise its cost and to increase the quality of the gathered information. Among the various means of collecting well-structured information with reasonably priced autonomous, vehicles that are equipped with on board sensing elements are considered to be one of the most promising technologies in the medium-term. However, the use of mobile robots in agricultural environments remains challenging as navigation in agricultural environments presents difficulties due to the variability and nature of the terrain and vegetation [8,9].

Research in navigation systems for agricultural applications has focused on guidance methods that employ global or local information. Guidance systems that use global information attempt to direct the vehicle along a previously calculated route based on a terrain map and the position of the vehicle relative to an absolute reference. In this case, global navigation satellite systems (GNSS), such as GPS, are usually employed. Guidance systems that utilise local information attempt to direct a vehicle based on the detection of local landmarks, such as planting patterns and intervals between crop rows.

The precision of the absolute positions that are derived from a GNSS can be enhanced by real-time kinematics (RTK), which is a differential GNSS technique that employs measurements of the phase of the signal carrier wave and relies on a single reference station or interpolated virtual station to provide real-time corrections and centimetre-level accuracy. The use of a RTK-GPS receiver as the only positioning sensor for the automatic steering system of agricultural vehicles has been examined in several previous studies [10,11]. Furthermore, recent studies [12,13] evaluate the use of low-cost GPS receivers for the autonomous guidance of agricultural tractors along straight trajectories.

Regardless of the type of GNSS used, this navigational technology has some limitations when the GNSS serves as the only position sensor for autonomous navigation of mobile robots. For this reason, RTK-GNSS is frequently combined with other sensors, such as inertial measurement units (IMUs) [14,15] or fibre-optic gyroscopes (FOGs) [16,17,18]. When a GNSS, even a RTK-GNSS, is employed as the only sensor for navigating across a crop without stepping on plants, an essential behaviour in crops such as maize, it is an indispensable requirement to perfectly know the layout of the crop rows and therefore, crops must be sowed with an RTK-GNSS-guided planting system or mapped using a georeferenced mapping technique. This approach is expensive and may not be feasible, which reduces the scope of the navigation systems that are based in GNSS. In this context, the proposed approach integrates a GNSS sensor with a camera (vision system) to obtain a robot’s behaviour, which enables it to autonomously cover an entire crop by following a previously established plan without stepping on the crop rows to avoiding damage to the plants. As discussed in the next section, the plan only considers the field contour and the crop type, which is readily available.

Vision sensors have been extensively utilised in mobile robot navigation guidance [19,20,21,22,23,24,25,26] due to their cost-effectiveness and ability to provide large amounts of information, which can also be employed in generating steering control signals for mobile robots. In addition, diverse approaches have been proposed for crop row detection. In previous studies [25,27], a segmentation step is applied to a colour image to obtain a binary image, in which white pixels symbolise the vegetation cover. Then, the binary image is divided into horizontal strips to address the perspective of the camera. For each strip, they review all columns of pixels. Columns with more white pixels than black pixels are labelled as potential crop rows, and all pixels in the column are set to white; otherwise, they are set to black. To determine the points that define crop rows, the geometric centre of the block with the largest number of adjacent white columns of the image is selected. Then, the method estimates the line defined by these points, which is based on the average values of their coordinates. Other approaches [28] transform an RGB colour image to grayscale and divide it into horizontal strips, where maximum grey values indicate the presence of a candidate row. Each maximum defines a row segment, and the centres of gravity of the segments are joined via a similar method to the centre of gravity that is utilised in the Hough transform or by applying linear regression. In [29], the original RGB image is transformed to a grayscale image and divided into horizontal strips. They construct a bandpass filter that is based on the finding that the intensity of the pixels across these strips exhibits a periodic variation due to the parallel crop rows. Sometimes, detection of the row is difficult as crops and weeds form a unique patch. The Hough transform [30] has been employed for the automatic guidance of agricultural vehicles [23,31,32,33]. Depending on the crop densities, several lines are feasible, and a posterior merging process is applied to lines with similar parameters [34,35,36]. When weeds are present and irregularly distributed, this process may cause failure detection. In [26,37], the authors employ stereo-images for crop row tracking to create an elevation map. However, stereo-based methods are only adequate when appreciable differences exist between the heights of crops and the heights of weeds, which is usually not the case when an herbicide treatment is performed in the field. In [38,39,40], crop rows are mapped under perspective projection onto an image that shows some behaviours in the frequency domain. In maize fields, where the experiments were performed, crops did not show a manifest frequency content in the Fourier space. In [41], authors analyse images that were captured from the perspective from a vision system that is installed onboard a vehicle and consider that the camera is being submitted to vibrations and undesired movements, which are produced as a result of vehicle movements on uneven ground. They propose a fuzzy clustering process to obtain a threshold to separate green plants or pixels (crops and weeds) from the remaining items in the field (soil and stones). Similar to other approaches, crop row detection applies a method that is based on image perspective projection, which searches for the maximum accumulation of segmented green pixels along straight alignments.

Regarding the type of vehicle, most studies of the autonomous guidance of agricultural vehicles have focused on tractors or heavy vehicles [10,12,13,15,17,20,21,22,23,24,26]. Moreover these large vehicles can autonomously navigate along the crop rows, but they are unable to autonomously cover an entire crop by performing the necessary manoeuvres for switching between crop rows. In other cases, the autonomous navigation is related to fleets of medium sized tractors able to carry weed control implements [42]. In PA, the use of small robots for inspecting an entire crop is a suitable choice over large machines to minimise the soil compaction. In this context, this study was conducted to support the use of small autonomous vehicles for inspection, with improved economic efficiency and reduced the impact on crops and soil compaction, which integrate both global location and vision sensors for obtaining a navigation system that enables the covering of an entire field without damage to the crop. Inspection based on small autonomous vehicles can be very useful for early pest detection by gathering geo-referenced information that is needed to construct accurate risk maps. More than one sampling is performed throughout the year, due to minimal crop impact, primarily if they can navigate across a field by following the crop rows, and soil compaction.

## 2. Materials and Methods

The robot used in this project is a commercial model (mBase-MR7 built by MoviRobotics, Albacete, Spain). It has four wheels with differential locomotion, no steering wheel and can rotate on its vertical axis. This work considered that the robot carries out the manoeuvres as if it could not rotate on its vertical axis, like other vehicles used within the agricultural fields (tractors, all-terrain vehicles, *etc.*). The reason is that often, in our experiments, the vehicle's wheels have dug up the land and the robot has gotten stuck. The on-board camera is a digital single-lens reflex camera (EOS 7D, Canon, Tokyo, Japan). The camera is located 80 cm aboveground at a pitch angle of 18° and is connected to an on-board computer (a Toughbook CF-19 laptop, Panasonic, Osaka, Japan equipped with an Intel Core i5 processor and 2 GB of DDR3 RAM) via a USB connector. The camera supplies approximately five frames per second, and each frame has a resolution of 1056 × 704 pixels. Other camera locations were studied, such as placing it facing down, focusing directly on the soil. However, this case only covered a small portion of terrain, and therefore the detection of the crop row was more vulnerable to local changes as sowing errors, weed patches, *etc.* Furthermore, the covered terrain was the area immediately in front of the robot, so when the robot needed to react to what it was present in the image, part of it had been left behind. Another analysed option was to place it ahead of the robot, in a forward position using a steel mast. However, this caused more vibrations on the camera during robot navigation, deteriorating the system operation significantly.

**Figure 1:**
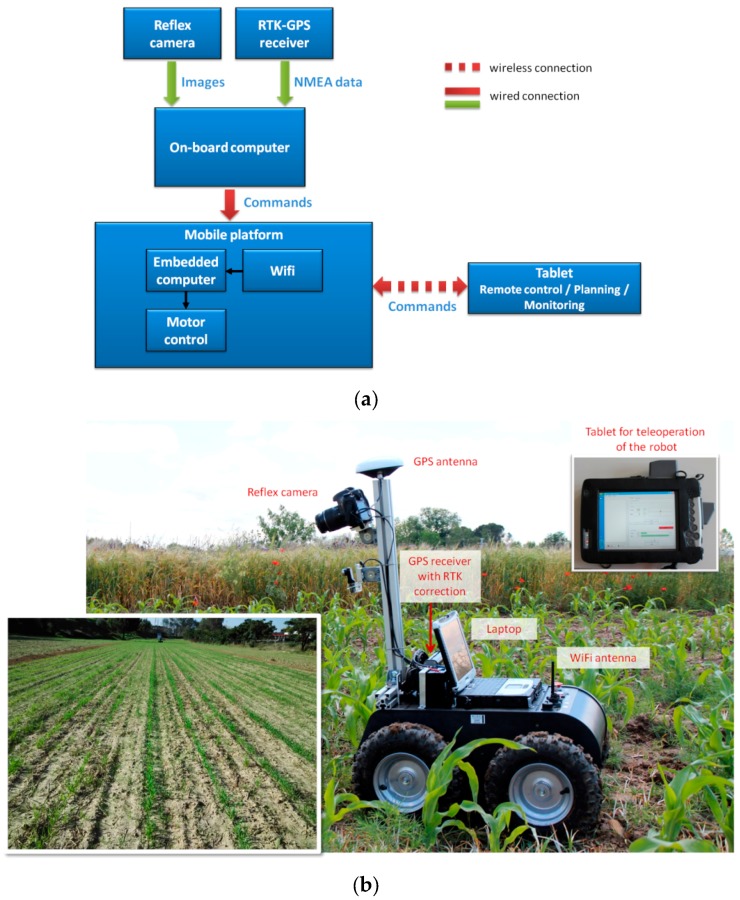
(**a**) The system architecture. (**b**) Devices integrated in the mBase-MR7 robot (right) and a maize crop field (left).

The vehicle equipment is complemented with a R220 GPS receiver (Hemisphere, Scottsdale, AZ, USA) with RTK correction for geo-referencing the gathered data and determining whether the robot has reached a field edge. A laptop (a tablet) is used to remotely control the robot. The architecture of the developed system is illustrated in Figure 1a, and photographs of the vehicle and maize crop rows in a field are shown in Figure 1b.

The plan to be followed by the robot is generated by a path planner. Path planning in agricultural fields is a complex task. Generally, it can be formulized as the well-known Capacited Vehicle Routing Problem (CVRP), as stated in [43]. Basically, the problem consists of determining the best inspection route that provides complete coverage of the field considering features such as the field shape, the crop row direction, the type of crop and some characteristics of the vehicles, such as the turning radii. The vehicles must completely traverse each row exactly once; therefore, the planner determines the order for performing the rows in such a manner that some optimisation criterion is minimal. Given a field contour, the planner can deduce the layout of the rows and the inter-row distance required by the plants, due to it assumed that the sowing was carried out by a mechanical tool that kept that distance in a reasonably precise way. In addition, a high precision is not required since the proposed platform and the proposed method exclusively use the trajectory points as guiding references to enter and leave the field rows.

**Figure 2:**
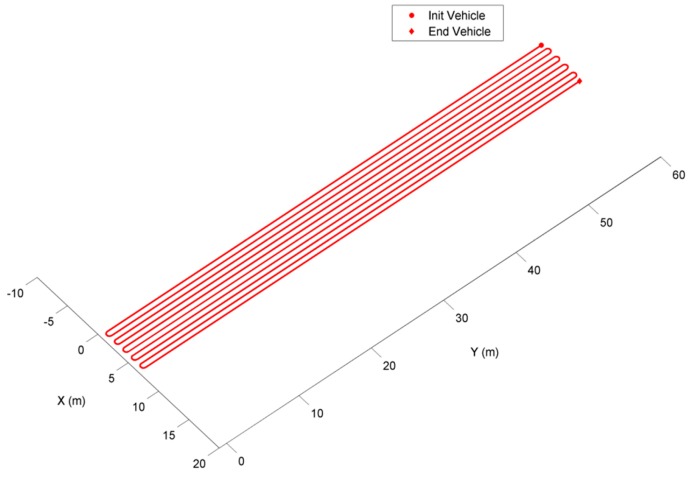
Path taken by the planner traversed all rows of the crop field in which the experiments were performed.

The planner employed in this work is described in [44] and uses a simulated annealing algorithm to address a simplified case of the general path planning problem with only one vehicle and considering the travelled distance as the optimisation criterion. Figure 2 shows the route that is generated by the planner for the crop field in which the experiments were performed. The field size was approximately 7 m × 60 m, which represents a total of ten crop rows in a maize planting schema with 0.7 m of distance between rows. In this case, the optimal trajectory was to sequentially explore the field, beginning at one edge and always going to an adjacent row as the vehicle is very small and has a turning radius that enables movement between adjacent rows despite the very small distances between rows. It is important to note that the system proposed in this study can work with any planner able to return the path to be followed as an ordered sequence of the number of pairs of GPS points as crop rows must be travelled, where the first pair of points represents the input point to the field to scout a row and the second pair point defines the field output point to go to the next row to be inspected.

To inspect a crop row, the robot is positioned at the beginning of the row, *i.e.*, in the approximate point established by the plan, with the row between its two front wheels. The robot advances, tracking the crop row using its on-board camera, until it reaches the end of the row. Once it has inspected a row, the robot executes the necessary manoeuvres to position itself at the head of the next row to be inspected; the approximate position of this row is also established in the plan. The process is repeated until all rows in the field have been inspected or when the plan has been completely executed. To achieve field inspection with complete coverage, a set of individual behaviours is required, including: (1) tracking of a crop row, (2) detection of the end of a crop row, and (3) transition to the head of the next row to be inspected (note that the plan provides only an approximate point). The proposed approaches to generate these behaviours in the robot are discussed in the following sections.

### 2.1. Crop Row Tracking Behaviour

**Figure 3:**
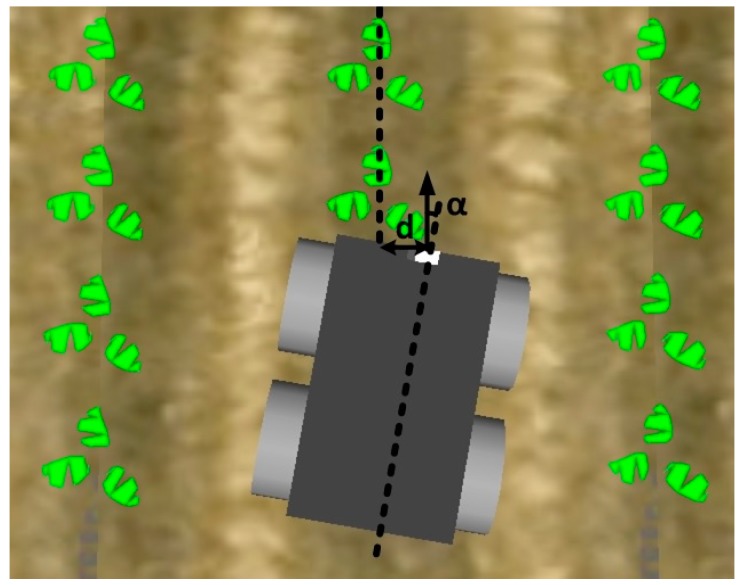
Pose of the robot with respect to the central crop row.

An image-processing method capable of extracting the layout of the crop rows in real time from images acquired with the camera positioned on the front of the robot was designed to allow the robot to use images of the crop rows to navigate. The purpose of the image processing is to obtain the vehicle’s position with respect to the crop row (see Figure 3), *i.e.*, the motion direction angle (*α*) and displacement or offset (*d*) between the robot centre and the closest point along the line defining the crop row.

The values of the vehicle’s offset (*d*) and angle (*α*) are provided to two fuzzy controllers, one for angular speed and one for linear speed, which determine the correction values for the steering to generate crop row tracking behaviour in the robot.

#### 2.1.1. Image Processing

In the row recognition process, the main problem is the identification of accurate features that are stable in different environmental conditions. The row detection process is accompanied by some difficulties, such as incomplete rows, missing plants, and irregular plant shapes and sizes within the row. In addition, the presence of weeds along the row may distort row recognition by adding noise to the row structure. The majority of the studies have focused on large agricultural vehicles, in which the displacement is more uniform than the displacement of small vehicles. In this study, the challenge is to robustly detect a crop row in the presence of weeds, despite the vibrations and variations in the camera, which are caused by the movement of a vehicle in the field. The majority of all methods for vegetation detection usually consider that all pixels that are associated with vegetation have a strong green component [45,46,47,48,49,50,51,52]. To take advantage of this characteristic, the utilisation of digital cameras in the visible spectrum and the use of the RGB colour model is frequent when working at the ground level [27,28,29,33,35,40,41,45,49,50,51,52]. The proposed row detection approach takes advantage of our previous study (refer to introduction) for designing and developing a real-time technique that properly works with RGB images that are acquired in varying environmental conditions.

**Figure 4:**
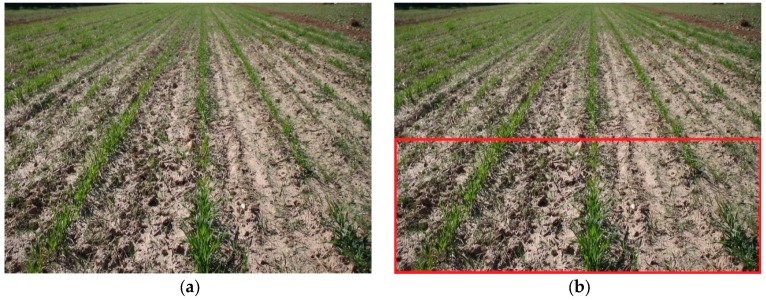
(**a**) A typical image acquired by the camera of the robot. (**b**) The working area of the image is delimited in red.

**Figure 5:**
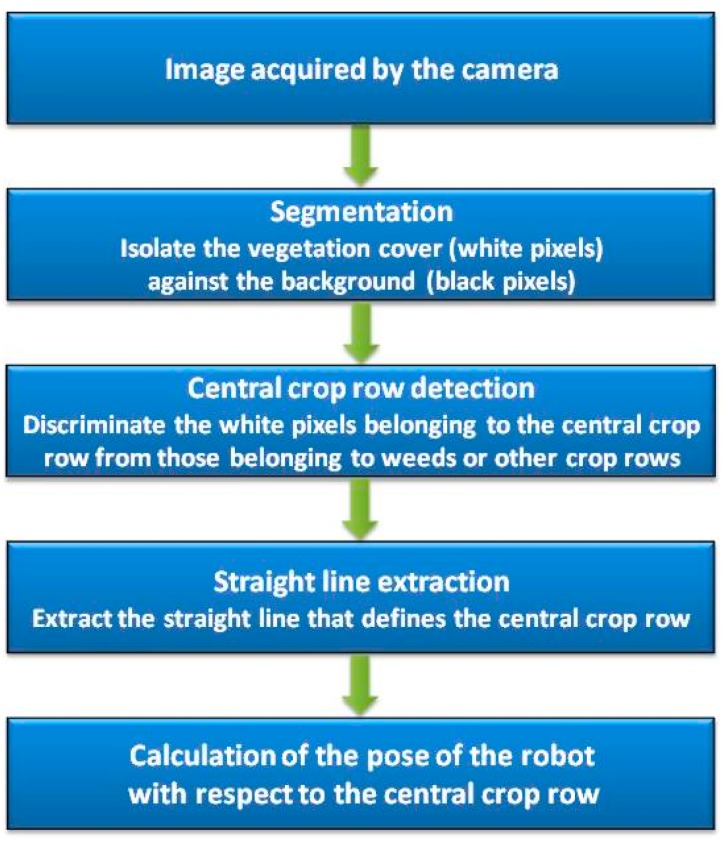
Diagram of the image processing phase.

A typical image acquired by the camera of the robot is shown in Figure 4a. In the upper corners of the image, the crop rows are difficult to distinguish due to the perspective in the image. To avoid these effects, the image was divided in half, and the upper half was discarded (Figure 4b). Thus, the image processing presented below only utilises the bottom half of each frame. Figure 5 shows a flowchart diagram of the image processing phase.

The objective of the first processing stage (segmentation stage) is to isolate the vegetation cover against the background, *i.e.*, to convert the input RGB image into a black-and-white image in which the white pixels represent the vegetation cover (weeds and crop) and the black pixels represent the remaining elements in the image (soil, stones, debris, straws, *etc.*).

Segmentation exploits the strong green components of the pixels representing vegetation. The coloured image can be transformed into a greyscale image by a linear combination of the red, green and blue planes, as shown in Equation (1):

(1) $$\begin{array}{c} \forall i\in rows\_image\wedge \forall j\in colums\_image:\\ Grey\left(i,j\right)=r*input_{red\left(i,j\right)}+g*input_{green\left(i,j\right)}+b*input\_blue\left(i,j\right) \end{array}$$

where *i* varies from 0 to 352, *j* from 0 to 1056, the *input_red(i, j)*, *input_green(i, j)*, *input_blue(i, j)* values are the non-normalised red, green, and blue intensities (0–255), respectively, at pixel (i, j) and *r*, *g*, *b* are the set of real coefficients that determine how the monochrome image is constructed. These values are crucial in the segmentation of vegetation against non-vegetation, and their selection is discussed in detail in [46,51]. In the proposed approach, the constant values were established to a set of values (r = −0.884, g = 1.262, and b = −0.311) that previously showed good results for similar images [53] compared with other well-known indices, such as *ExG* (r = −l, g = 2, b = −1) [46].

**Figure 6:**
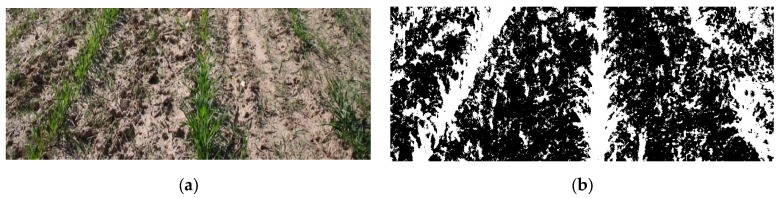
(**a**) Original image with marked weed presence. (**b**) Segmented image.

In the next step, a threshold is used to convert the monochrome greyscale image into a binary image in which the white pixels represent vegetation and the black pixels non-vegetation. The threshold depends on the lighting conditions. Therefore, the threshold is not fixed in the approach outlined here but is instead calculated for each analysed image as the mean value of the grey intensities in the image. The results of the segmentation stage are illustrated in Figure 6.

The goal of the next stage (central crop row detection), which processes the binary images obtained in the previous stage, is to discriminate the white pixels belonging to the central crop row from those belonging to weeds or other crop rows. To achieve this goal, the method developed based on [54] first performs a morphological opening operation (erosion followed by dilation) of the binary image to eliminate isolated white pixels and highlight areas with a high density of white pixels. One of the aims of this operation is to eliminate the small groups of black pixels that appear inside the crops. The structural element used for the dilation and erosion is a 3 × 3 square. The borders of the resulting image are then extracted using the Sobel operator such that all pixels in the transitions (white to black and vice versa) are marked. The image is then divided into three horizontal strips to deal with perspective. Each strip is processed independently using the following methods.

The potential centre of the central crop row is the column of the strip with the greatest number of white pixels within a search window. To identify this column, a vector is built using the same number of components as the size of the window, where each component stores the number of white pixels (vegetation) of the associated column. The perspective of the original images is also considered when defining this window; thus, the size of the window varies depending on the proximity of the camera to the analysed strip (Thales’ intercept theorem). The search window is centred in the middle of the image in the first frame, but due to overlap between subsequent frames (the robot advances 6 cm between frames at its highest speed), the possible centre of the row in the next frame is searched around the central position identified in the previous frame.

After the potential centre of the row is identified, the algorithm begins to identify the edges delimiting the crop row, searching to the right and to the left from the centre found. To confirm that a crop edge has been reached, the method uses three pixel labels: white, black and border. When the pixel encountered is white, it is marked as belonging to the crop row, and the algorithm continues with the next pixel. When a border pixel is located, the exploration has reached either a crop edge or a group of black pixels inside the crop. The distance to the next border pixel can be used to distinguish between these two cases. The distance to the next crop row or to a weed between crop rows is greater in the former case than in the latter, *i.e.*, inside the crop row. In fact, two distance thresholds are established, $D_{1}\leq D_{2}$, such that if the computed distance is greater than threshold $D_{2}$, the exploration has reached a crop edge, whereas if the distance is less than $D_{1}$, a group of black pixels inside the crop has been reached. If the distance is between $D_{1}$ and $D_{2}$, the method uses the previously generated vector to locate the centre of the row and proceeds as follows. The percentage of white pixels in each column is calculated for the range of components of the vector between the current position of the pixel and the position of the edge. If this percentage is higher than a threshold called *min_proportion*, the algorithm indicates that it has reached a group of black pixels inside the crop because the column to which this group of black pixels belongs has a large number of white pixels because it is part of a crop row. If it is lower than this threshold, the algorithm indicates that it has reached the edge of the crop row because the number of black pixels in the columns that separate it from the next crop row or weed is large.

This procedure is formally set out in Table 1, where *p* is the pixel currently being explored, *n* is the next (not black) pixel in the processing order at a distance *d*, and $D_{1}$, $D_{2}$ and *min_proportion* are the three parameters of the method.

**Figure 7:**
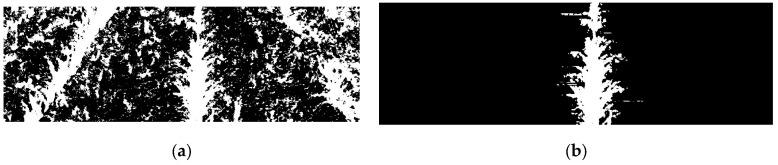
(**a**) Segmented image. (**b**) Central crop row detected by applying the proposed detection method to Figure 6a.

Due to the effects of perspective in the image, the width of the crop rows varies depending on proximity to the camera. This phenomenon is considered in the method. The parameter $D_{1}$ varies between 5 and 10, starting at 5 when the farthest strip from the camera is analysed and reaching 10 when analysing the closest strip. Likewise, the parameter $D_{2}$ varies between 10 and 20. The value of the threshold *min_proportion* is 0.6. Using this process, the central crop row can be detected from the binary image in the presence or absence of weeds (see Figure 7).

**Figure 8:**
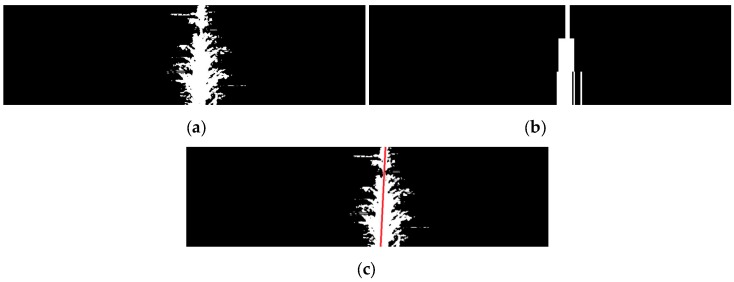
(**a**) Central crop row obtained in the previous stage. (**b**) White and black columns defined by the algorithm from Figure 8a. (**c**) Straight line defining the crop row centre in Figure 8a.

After the central crop row is detected, the purpose of the next stage is to extract the straight line that defines the central crop row from the image resulting from the last stage. To address the slight perspective of the camera, the image is divided into three horizontal strips, which are processed to obtain three points to define the central crop row. The previous stage assumed that the potential centre of the central crop row was the column with the greatest number of white pixels. However, this may not be the case when groups of black pixels are present inside the crop. In the previous stage, such an occurrence does not affect the algorithm, that is, it does not matter whether the algorithm starts in the exact centre of the crop row as long as it is located inside the row. However, the extraction of the straight line that defines the crop row requires that the centre of the crop row be located as accurately as possible. Thus, a search window is defined in the same way as in the previous stage, and a vector is obtained with as many components as the window size, where each component stores the number of white pixels of the associated column. Next, the maximum value of the vector is computed, and all columns in the image whose vector component is greater than 80% of this maximum value are converted to white, whereas the rest are converted to black (Figure 8a). To determine the points that define the central crop row, in each strip, the geometric centre of the block with the largest number of white columns together is chosen. The algorithm then estimates the straight line defining the three centres identified (one for each strip) using the least squares method (Figure 8b). If less than two centres are located (*i.e.*, sowing errors), the algorithm employs the straight line that is obtained in the previous frame. After obtaining the straight line that defines the crop row, the angle (α) between the direction of motion of the robot and the centre line of the crop row and the displacement (d) between the centre of the vehicle and the centre of the crop row are calculated (refer to Figure 3).

#### 2.1.2. Navigation Control

Many approaches exist that address the actuator control of a car. Conventional control methods produce reasonable results at the expense of high computational and design costs because obtaining a mathematical model of the vehicle becomes extremely expensive [55,56], since wheeled mobile robots are characterised by nonlinear dynamics and are affected by an important number of disturbances, such as turning and static friction or variations in the amount of cargo. Alternatively, we can approach human behaviour for speed and steering control using artificial intelligence techniques, such as neural networks [57]. However, the technique that provides a better approximation to human reasoning and gives a more intuitive control structure is the fuzzy logic [58,59]. Some authors have proposed solutions that are based in fuzzy logic for autonomous navigation [60,61,62,63,64], which demonstrates their robustness. In [60], fuzzy control is employed in a real car to perform trajectory tracking and obstacle avoidance in real outdoor and partially known environments. In [61], fuzzy controllers are implemented in a real car to conduct experiments on real roads within a private circuit. Their results show that the fuzzy controllers perfectly mimic human driving behaviour in driving and route tracking, as well as complex, multiple-vehicle manoeuvres, such as adaptive cruise control or overtaking. In [62], the navigation of multiple mobile robots in the presence of static and moving obstacles that employ different fuzzy controllers is discussed. Their experiments demonstrate that robots are capable of avoiding obstacles and negotiating dead ends, as well as efficiently attaining targets. In [63], authors develop and implement fuzzy controllers for the steering and speed control of an autonomous guided vehicle. Their results indicate that the proposed controllers are insensitive to parametric uncertainty and load fluctuations and outperformed conventional proportional-integral-derivative (PID) controllers, particularly in tracking accuracy, steady-state error, control chatter and robustness. In [64], an unknown path-tracking approach is addressed, based on a fuzzy-logic set of rules, which emulates the behaviour of a human driver. The method applies approximate knowledge about the curvature of the path ahead of the vehicle and the distance between the vehicle and the next turn to attain the maximum value of the linear velocity that is required by the vehicle to safely drive on the path.

**Figure 9:**
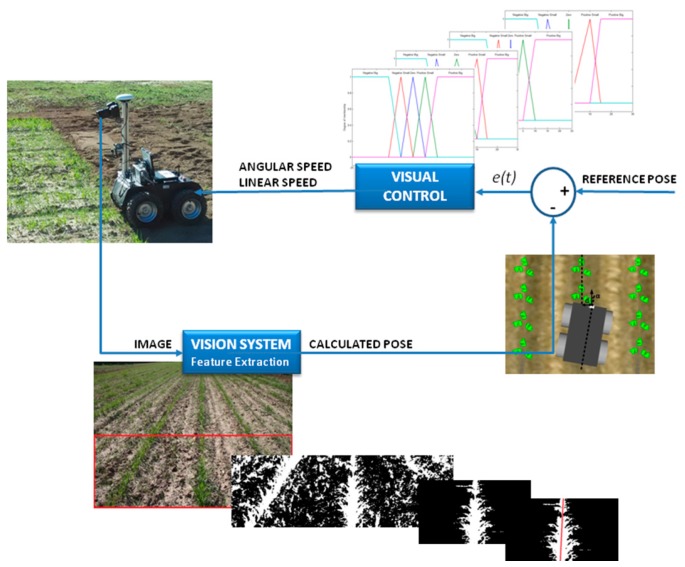
Visual scheme of the developed control.

The proposed navigation control that enables a robot to follow crop rows comprises two fuzzy controllers (Figure 9): one for angular speed and one for linear speed. Both controllers are fuzzy and therefore imitate the behaviour of a skilled driver; for example, if the vehicle is moved to one side of the crop row to be tracked, the robot must correct its position in the other direction such that it navigates with the crop row between its wheels.

The inputs of the controller acting on the angular speed of the robot are the displacement of the centre of the vehicle from the midpoint of the crop row (*d* in Figure 3) and the angle of orientation of the robot (*α* in Figure 3). The controller produces the angular speed of the vehicle as an output. The rules used in the controller take the following form:

If (Offset is Negative Big) and (Angle is Positive Small) then (Angular Speed is Positive Small)

These rules are summarised in Table 2.

**Figure 10:**
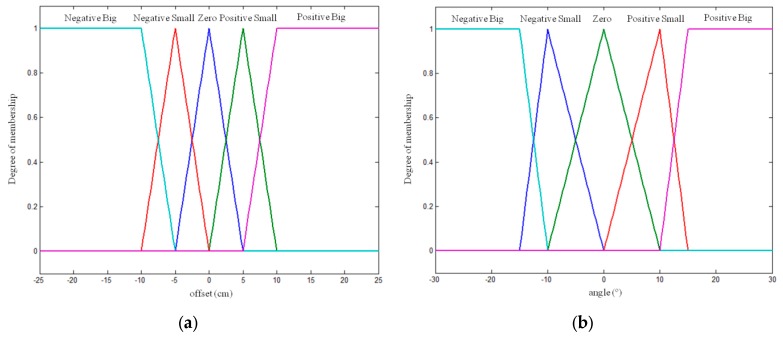
Angular speed controller. Fuzzy sets of input variables: (**a**) offset and (**b**) angle.

Negative_Big, Negative_Small, Zero, Positive_Small and Positive_Big are the fuzzy sets shown in Figure 10 and are determined in consonance with the features of the robot. The value ranges of each set in the input corresponding to the offset of the robot regarding the midpoint of the crop row (*d*) were selected based on the distance between the wheels of the robot (41 cm). In the case of the angle of orientation (*α*), to select the value ranges of each set, it was assumed that the robot would crush the crop row if it turned at an angle of 30° or −30°.

The Takagi-Sugeno implication is compatible with applications that require on-time responses [58] and is therefore used in this work. The output of the controller is the singleton-type membership functions shown in Figure 12a. The range of angular speeds allowed by the robot is −90 °/s to 90 °/s, and the fuzzy sets are chosen to cover the speed range necessary to control the robot smoothly. The input variables in the controller of the linear speed of the robot are the same as the previous controller, *i.e.*, offset and angle, and the fuzzy rules defined are summarised in Table 3.

**Figure 11:**
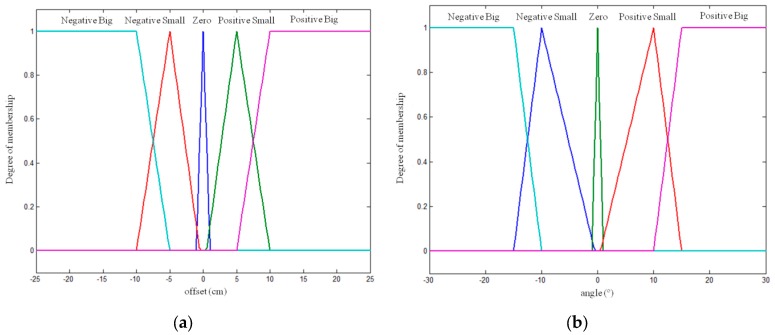
Linear speed controller. Fuzzy sets of input variables: (**a**) offset and (**b**) angle.

The value ranges of the fuzzy sets (Figure 11) for both displacement and angle are selected so that the vehicle moves at maximum speed (30 cm/s) provided that it is correctly positioned; otherwise, it slows to avoid treading on the crop row and crushing the crop.

**Figure 12:**
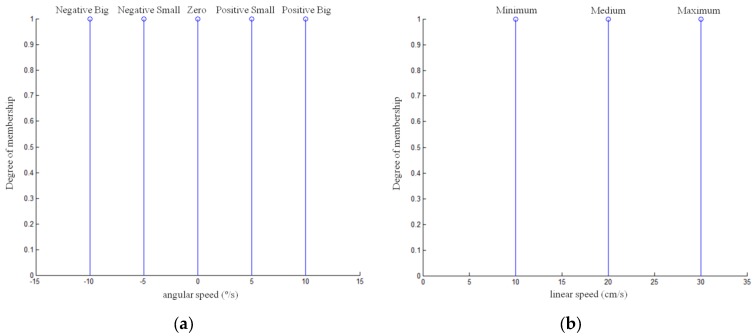
Fuzzy sets for the output variables: (**a**) angular speed and (**b**) linear speed.

The controller output is the linear speed applied to the vehicle and is characterised by three fuzzy sets (MIN: minimum, MED: medium, MAX: maximum), whose values are selected to cover the robot’s range of allowed speeds (Figure 12).

### 2.2. End of Crop Row Detection

**Figure 13:**
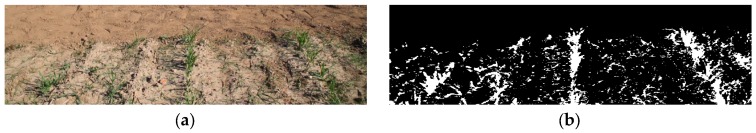
(**a**) Image acquired by the robot of the end of a crop row. (**b**) Segmented image of Figure 13a. The number of pixels that is associated with vegetation (white pixels) is less than 80% of the number of pixels in the reference image (Figure 6), and the robot location is very close to the output point that is defined in the plan for the row that is being scouted. Thus, the robot determines that it has reached the end of the crop row.

To complete tracking of the inspected crop row, the robot must detect the end of the crop row (Figure 13). For this purpose, the number of pixels in the image belonging to vegetation is used. When the robot begins to track a crop row, the number of pixels belonging to vegetation in the first image of the tracking is stored as a reference value. As the robot advances, it detects the number of pixels that are associated with the vegetation in each image. When this number is less than 80% (threshold was determined using the trial and error method) of the reference value, the robot assumes that it has reached the end of the crop row and checks with the on board GPS to ensure that this position is consistent with the output point given in the plan (with a margin of error). If they are consistent, the robot stops; otherwise, it continues tracking the crop row using the direction of the central row that is obtained in the previous frame. The loss of pixels that are associated with vegetation is consistent with a possible sowing error.

### 2.3. Row Change Behaviour

**Figure 14:**
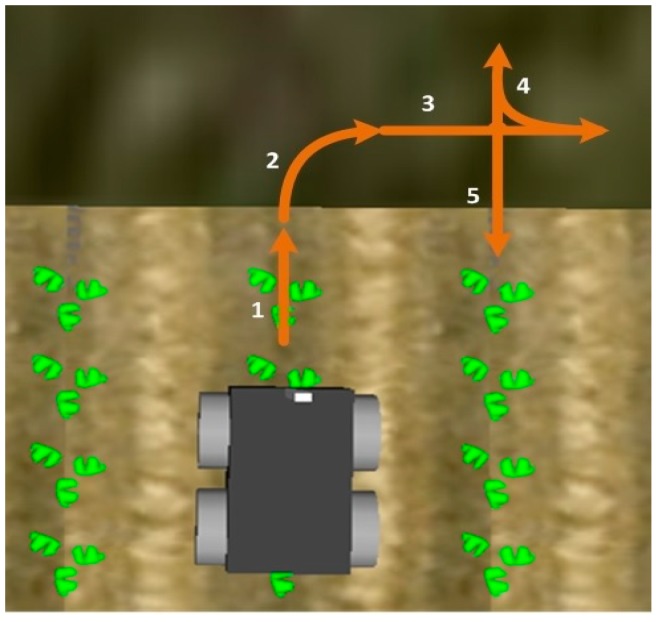
Manoeuvres defined for crop row change.

After detecting the end of a crop row, the row change behaviour is performed. In this behaviour, the robot performs the necessary manoeuvres to position itself at the head of the next row to be inspected. This set of manoeuvres consists of a combination of straight-line and circular-arc movements with the help of the on board GPS. Specifically, it is a sequence of the following five moves (Figure 14): (1) straight-line forward movement to position itself outside the crop, in the header of the crop, to avoid crushing the crop in subsequent manoeuvres; (2) circular-arc movement towards the corresponding side of the next crop row to be inspected to position itself perpendicular to the crop rows; (3) straight-line forward movement; (4) reverse circular-arc movement away from the crop row to position itself parallel to the direction of the crop rows; and (5) straight-line forward movement to place itself at the head of the next crop row to be inspected according to the plan.

## 3. Results and Discussion

**Figure 15:**
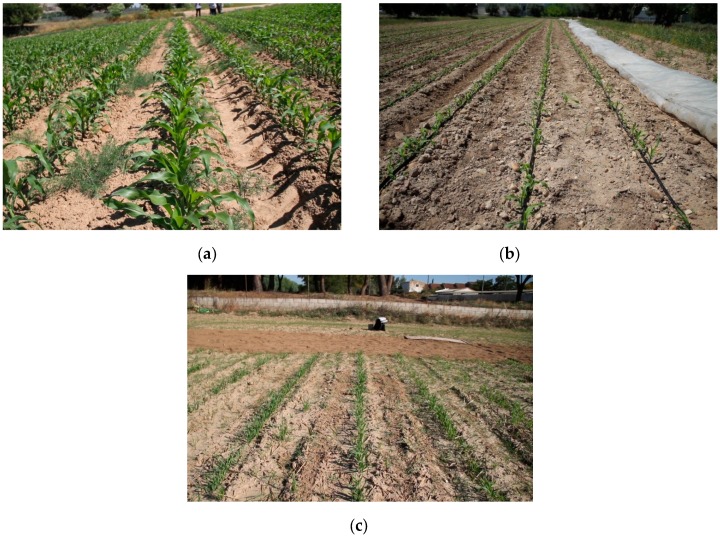
(**a**)–(**c**) are examples of different images that were acquired by the camera of the robot. (**a**) shows the maize crops; (**b**) shows the crop rows where the irrigation system used is visible; (**c**) shows the kind of image to be taken when a crop border is being reaching.

To evaluate the performance and robustness of the proposed approach for central row detection, a set of 500 images, which were acquired by the robot working in remote control mode in different maize fields and distinct days at Arganda del Rey (Madrid, Spain), were utilised. The images were captured in different conditions of illumination, growth stages, weed densities and camera orientations; the robot operates in these conditions when it autonomously navigates. Figure 15 illustrates several examples of the employed images.

The proposed approach was compared with a detection method that is based on the Hough transform [30], which is a strategy that was successfully integrated in some of our previous studies [36] in terms of effectiveness and processing time. The last aspect is really important in cases, such as this case, in which real-time detection is required. Both methods analysed the bottom half of each image, *i.e.*, 1056 × 352 pixels. The effectiveness was measured based on an expert criterion, in which the line that was detected was considered to be correct when it matched the real direction of the crop row. Table 4 shows the results from processing the 500 images with both approaches. The performance of the proposed approach exceeds the performance of a Hough-transform based strategy by 10%. The processing time that is required by the proposed approach is approximately four times less than the processing time required for the Hough transform, which indicates that the proposed approach can process approximately 14 frames in the best case compared with the Hough transform approach. The Hough transform approach can process three frames per second, which is less than the five frames per second that the EOS 7D camera provides (refer to Section 2 of this paper).

**Figure 16:**
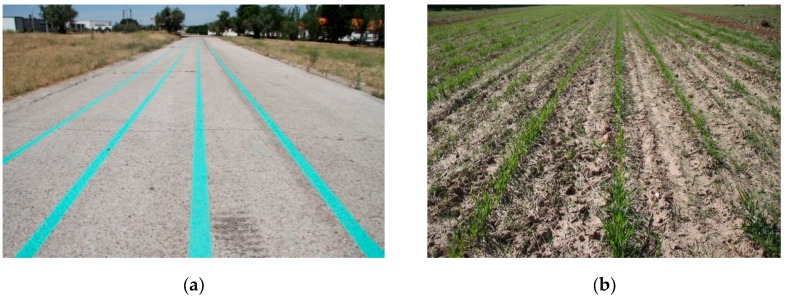
(**a**) First test environment. (**b**) Crop employed in the experiments.

To test the different behaviours developed for the robot, a test environment was established. Green lines were painted in an outside soil plane to simulate crop rows. The lines were 30 m in length and spaced 70 cm apart, as illustrated in Figure 16a. To make the environment more realistic, weeds and sowing errors were introduced along the lines.

**Figure 17:**
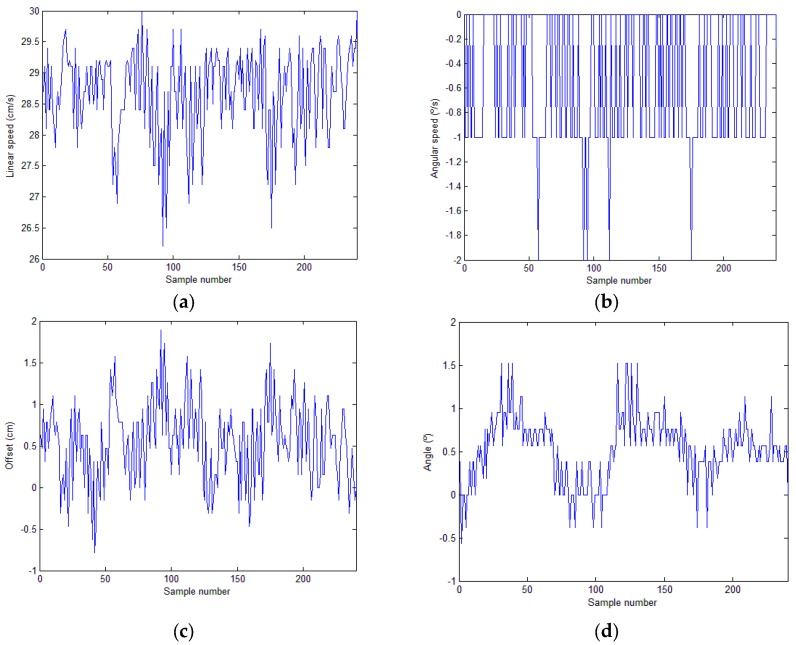
First test environment: (**a**) Evolution of the linear speed of the robot. (**b**) Evolution of the angular speed of the robot. (**c**) Evolution of the offset of the robot relative to the line. (**d**) Evolution of the angle of the robot relative to the line.

In this test environment, several experiments were performed to verify the proper functioning of the three autonomous robot behaviours described above: tracking, detection of the end of the crop row and row change. During the experiments, the robot followed the lines without headings, detected the row end, and changed rows, continuing the inspection, without human intervention. Figure 17 shows the evolution of the linear and angular speeds of the robot during the inspection of a line and the position of the robot with respect to the line (offset and angle) extracted by the image algorithm. Table 5 shows the mean, standard deviation, minimum and maximum of the linear speed, angular speed, offset and angle. The robot adjusted its linear speed depending on the error in its position; the speed was highest when the error in its position was 0 and decreased as the error increased, confirming the proper operation of the fuzzy controller of the linear speed. Variations in the angular speed were minimal because the error in the position was very small (offset and angle). The slight corrections to the left in the angular speed (negative values) were due to the tendency of the robot to veer towards the right when moving forward in its working operation (remote control mode). The design of the controller allowed these corrections to be made softly and imperceptibly during robot navigation and yielded movement in a straight line, which is impossible in remote control mode.

**Figure 18:**
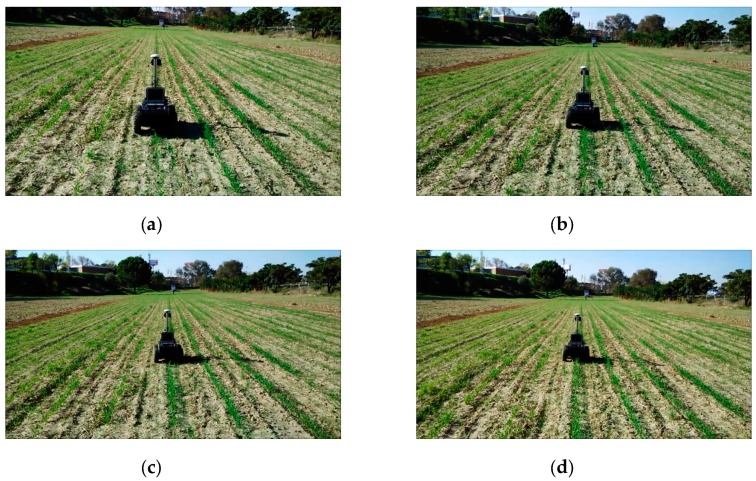
Crop row tracking behaviour. Images from (**a**) to (**d**) show a sequence of different frames of the video available in [65].

**Figure 19:**
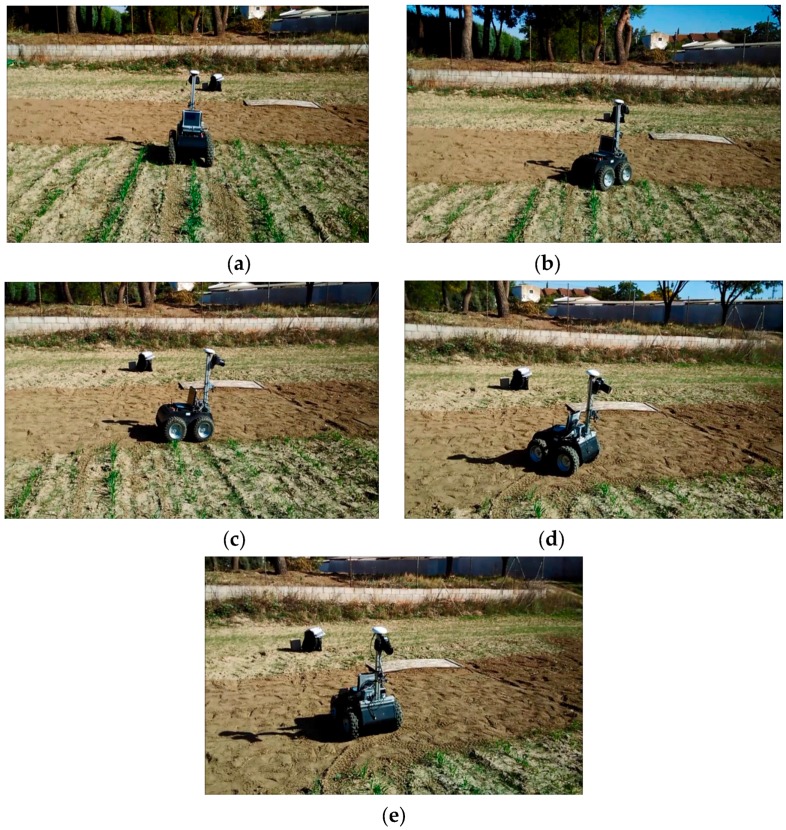
Sequence of manoeuvres performed by the robot to change rows. (**a**) corresponds with manoeuvre 1 in Figure 14, *i.e.*, the straight-line forward movement; (**b**) with the circular-arc movement, manoeuvre 2; (**c**) with straight-line forward movement, manoeuvre 3; (**d**) with reverse circular-arc movement, manoeuvre 4; (**e**) with straight-line forward movement, manoeuvre 5. Video can be found in [65].

After confirming the performance of the robot in the test environment, several experiments were conducted in a real field at Arganda del Rey (Madrid, Spain). The field was a cereal field with crop row spacing of 70 cm, which resembles a maize sowing schema. The rows were not perfectly straight and were characterised by strong high weed presence, as illustrated in Figure 16b. The robot was tested in different crop rows that covered the entire field. In this environment, the robot again followed the crop rows without crushing the crops (Figure 18), detected the row end, and performed the necessary manoeuvres to change rows (Figure 19) to continue the inspection without human intervention.

**Figure 20:**
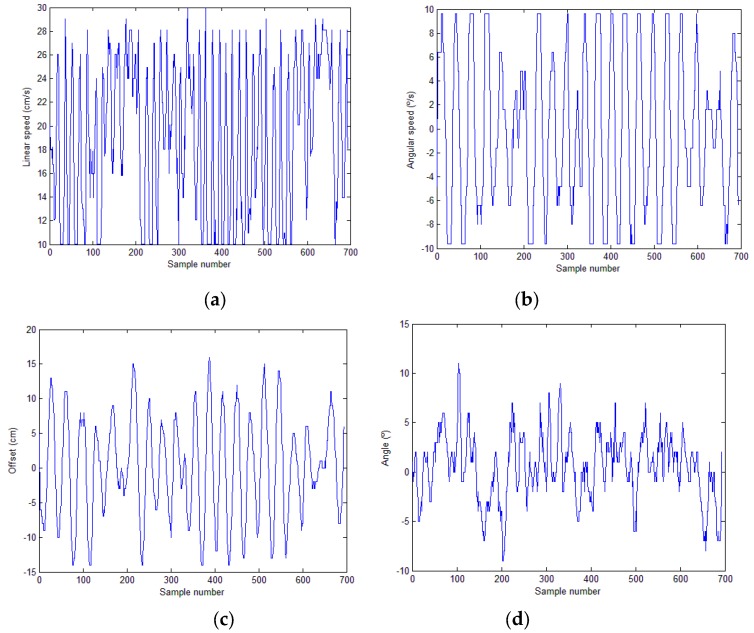
Tests in a real field: (**a**) Evolution of the linear speed of the robot. (**b**) Evolution of the angular speed of the robot. (**c**) Evolution of the offset of the robot relative to the line. (**d**) Evolution of the angle of the robot relative to the line.

However, the crop row tracking performance was worse in the real environment compared to the first test environment. Figure 20 shows the evolution of the linear and angular speeds of the robot during the inspection of a line and the position of the robot with respect to the line (offset and angle) extracted by the image algorithm. Table 6 presents the values (mean, standard deviation, minimum and maximum) obtained for linear speed, angular speed, offset and angle. The difference in performance relative to the first environment is evident. The results were a consequence of the estimated position of the robot relative to the crop row (offset and angle). The image-processing algorithm correctly extracted the straight line defining the crop row. In contrast to the first test environment, the rows were not perfectly straight, which affected the performance of the robot. However, the difference in performance between the environments was primarily due to the differences between the flat soil of the first environment and the abrupt soil of the field. The irregularities and roughness of the field, which hindered the movement of the robot, and the features of the vehicle, which did not provide any damping, caused vibrations and swinging in the pitch, yaw and roll angles of the camera during robot navigation, particularly on the most rugged soil. These effects increased the variation in the estimated position of the robot as it moved in the field, which decreased the performance of the robot, slowed row tracking, and induced headings in stretches where the wheels of the robot had poor traction.

The observed variations are one of the challenges of using small field inspection vehicles instead of tractors or large machines; for the latter, the displacement is more uniform, with fewer vibrations and variations in the camera. As observed in the experiments, although the robot was able to navigate along the crop rows without crushing them, to improve its performance, the mechanical features of the robot must be improved, or a more suitable vehicle must be adopted to navigate the crop field. Note that the proposed navigation system can be easily adapted to control other types of vehicles as it is based on driver behaviour rather than the vehicle model.

## 4. Conclusions

Crop inspection is a very important task in agriculture and PA, particularly when information about weed distribution is essential for generating proper risk maps that aid in site-specific treatment tasks. Among the various means of collecting field information, the use of small, autonomous vehicles with on-board sensing elements to minimise the impact on crop and soil compaction is quite promising. An autonomous field inspection vehicle, which autonomously navigates by visually tracking crop rows and following a previously established plan that guarantees the entire coverage of a field, was presented in this study.

A set of basic behaviours necessary for an autonomous mobile robot to inspect a crop field with full coverage was established and implemented, including the tracking of a crop row, the detection of the end of a crop row, and the correct positioning of the robot at the head of the next row.

An image-processing method capable of extracting the central crop row in the presence of weeds under uncontrolled lighting conditions in real time from images acquired with a reflex camera positioned at the front of the robot was developed. Two fuzzy controllers were also designed to achieve visual servoing for robot navigation. A method for detecting the end of the crop row using the images acquired by the camera was developed. In addition, the manoeuvres required to change rows were implemented.

Several experiments were conducted to test the performance of the proposed and developed behaviours. These behaviours were performed in a test environment with plane soil. The robot was able to follow the lines, detect the row end, and change lines to continue the inspection without human intervention. The good results are due to the very small errors in the estimated position of the robot relative to the crop row.

After these tests, a set of trials was performed on a real crop field. The robot was able to perform its behaviours correctly, although with reduced performance compared to the first test environment. This decreased performance is due to the variations in the estimated position of the robot relative to the crop row. The irregularities and roughness of the field hindered the movement of the robot, which lacked damping features, resulting in camera vibrations and swinging in its pitch, yaw and roll angles during robot navigation, particularly in the most rugged soil. These effects slowed the tracking of the crop row and induced headings in some areas where the wheels of the robot had poor traction.

These variations were the main disadvantage we encountered when working with this small vehicle in the field. To improve the performance of the developed behaviours, the mechanical features of the robot should be modified, or an alternative vehicle better suited to navigating a crop field should be adopted. In the latter case, the approaches that are proposed in this paper can be integrated in the new vehicle just adapting slightly the value ranges of the fuzzy sets of the navigation control to the new vehicle.

## Figures and Tables

**Table 1:** Crop row detection method.

Type of Current Pixel	Distance *d* (in pixels) until next non-Black Pixel		
	$d\leq D_{1}$	$D_{1}<d\leq D_{2}$	$d>D_{2}$
White	Mark all pixels from *p* to *n* and jump to n (*p*←*n*)	Mark all pixels from *p* to *n* and jump to *n* (*p*←n*)*	Stops
Border	Mark all pixels from *p* to *n* and jump to *n* (*p*←*n*)	IF White pixels(input(1…N), *p*…*n*)) > *min_proportion* THEN Mark all pixels from *p* to *n* and jump to *n* (*p* = *n*) ELSE Stops	Stops
Black	Jump to *n* (*p*←*n*)	Jump to *n* (*p*←*n*)	Stops

**Table 2:** Fuzzy control rules for angular speed. The fuzzy labels in this table correspond to the fuzzy sets shown in Figure 10. Furthermore, Figure 11 shows the fuzzy set for linear speed used in Table 3 whereas fuzzy sets for the output variables are illustrated in Figure 12.

	Offset *d*	Negative Big	Negative Small	Zero	Positive Small	Positive Big
Angle *α*						
**Negative Big**		Positive Big	Positive Big	Positive Big	Positive Small	Zero
**Negative Small**		Positive Big	Positive Small	Positive Small	Zero	Negative Small
**Zero**		Positive Big	Positive Small	Zero	Negative Small	Negative Big
**Positive Small**		Positive Small	Zero	Negative Small	Negative Small	Negative Big
**Positive Big**		Zero	Negative Small	Negative Big	Negative Big	Negative Big

**Table 3:** Fuzzy control rules for linear speed. The fuzzy labels in this table correspond to the fuzzy sets shown in Figure 11 and Figure 12b.

	Offset *d*	Negative Big	Negative Small	Zero	Positive Small	Positive Big
Angle α						
**Negative Big**		Minimum	Minimum	Minimum	Minimum	Minimum
**Negative Small**		Minimum	Minimum	Medium	Medium	Medium
**Zero**		Minimum	Medium	Maximum	Medium	Minimum
**Positive Small**		Medium	Medium	Medium	Minimum	Minimum
**Positive Big**		Minimum	Minimum	Minimum	Minimum	Minimum

**Table 4:** Detection of the central crop row. Performance of the proposed approach and an approach that is based on the Hough transform.

Approach	Effectiveness (%)	Mean Processing Time (seconds)
Proposed approach	96.4	0.069
Hough-transform-based approach	88.4	0.258

**Table 5:** Results obtained in the test environment.

Test environment	Mean	Std. dev.	Minimum	Maximum
**Linear speed (cm/s)**	28.66	0.68	26.20	30.00
**Angular speed (°/s)**	−0.57	0.53	−2.00	0.00
**Offset (cm)**	0.55	0.47	−0.78	1.88
**Angle (°)**	0.52	0.38	−0.56	1.51

**Table 6:** Results obtained in a real field.

Real Field	Mean	Std. dev.	Minimum	Maximum
**Linear speed (cm/s)**	18.87	6.16	10.04	29.94
**Angular speed (°/s)**	−0.14	6.40	−9.60	9.60
**Offset (cm)**	0.05	7.33	−14.00	16.00
**Angle (°)**	0.53	3.32	−9.00	11.00

## Abbreviations

The following abbreviations are used in this manuscript:

CVRP: Capacited Vehicle Routing Problem
DSS: Decision Support System
FOG: Fibre Optic Gyroscope
GIS: Geographical Information System
GNSS: Global Navigation Satellite System
IMU: Inertial Measurement Unit
PA: Precision Agriculture
PID: Proportional Integral Derivative
RTK: Real Time Kinematic
ExG: Excess green index
*b*: coefficient of the blue plane
*d*: distance
*g*: coefficient of the green plane
*min_proportion*: Percentage threshold
*r*: coefficient of the red plane
*α*: motion direction angle
$D_{1}:$: Lower distance threshold
$D_{2}$: Upper distance threshold

## References

[1] Gebbers R., Adamchuk V.I. (2010). Precision agriculture and food security. Science.

[2] Srinivasan A. (2006). Handbook of Precision Agriculture: Principles and Applications.

[3] Stafford J.V. (2000). Implementing precision agriculture in the 21st century. J. Agric. Eng. Res..

[4] Reid J.F. (2011). The impact of mechanization on agriculture. Bridge Agric. Inf. Technol..

[5] Senay G.B., Ward A.D., Lyon J.G., Fausey N.R., Nokes S.E. (1998). Manipulation of high spatial resolution aircraft remote sensing data for use in site-specific farming. Trans. ASAE.

[6] Rew L.J., Cousens R.D. (2001). Spatial distribution of weeds in arable crops: are current sampling and analytical methods appropriate?. Weed Res..

[7] Marshall E.J.P. (1988). Field-scale estimates of grass weed populations in arable land. Weed Res..

[8] Slaughter D.C., Giles D.K., Downey D. (2008). Autonomous robotic weed control systems: A review. Comput. Electron. Agric..

[9] Li M., Imou K., Wakabayashi K., Yokoyama S. (2009). Review of research on agricultural vehicle autonomous guidance. Int. J. Agric. Biol. Eng..

[10] Stoll A., Kutzbach H.D. (2000). Guidance of a forage harvester with GPS. Precis. Agric..

[11] Blackmore B.S., Griepentrog H.W., Nielsen H., Nørremark M., Resting-Jeppesen J. Development of a deterministic autonomous tractor. Proceedings of the 2004 CIGR Olympics of Agricultural Engineering.

[12] Gomez-Gil J., Alonso-Garcia S., Gómez-Gil F.J., Stombaugh T. (2011). A simple method to improve autonomous GPS positioning for tractors. Sensors.

[13] Alonso-Garcia S., Gomez-Gil J., Arribas J.I. (2011). Evaluation of the use of low-cost GPS receivers in the autonomous guidance of agricultural tractors. Spanish J. Agric. Res..

[14] Eaton R., Katupitiya J., Siew K.W., Howarth B. (2010). Autonomous farming: Modelling and control of agricultural machinery in a unified framework. Int. J. Intel. Syst. Technol. Appl..

[15] Kise M., Noguchi N., Ishii K., Terao H. The development of the autonomous tractor with steering controller applied by optimal control. Proceeding of the 2002 Automation Technology for Off-Road Equipment.

[16] Nagasaka Y., Umeda N., Kanetai Y., Taniwaki K., Sasaki Y. (2004). Autonomous guidance for rice transplanting using global positioning and gyroscopes. Comput. Electron. Agric..

[17] Noguchi N., Kise M., Ishii K., Terao H. Field automation using robot tractor. Proceeding of the 2002 Automation Technology for Off-Road Equipment.

[18] Bak T., Jakobsen H. (2004). Agricultural robotic platform with four wheel steering for weed detection. Biosyst. Eng..

[19] Marchant J.A., Hague T., Tillett N.D. (1997). Row-following accuracy of an autonomous vision-guided agricultural vehicle. Comput. Electron. Agric..

[20] Billingsley J., Schoenfisch M. (1997). The successful development of a vision guidance system for agriculture. Comput. Electron. Agric..

[21] Slaughter D.C., Chen P., Curley R.G. (1999). Vision guided precision cultivation. Precis. Agric..

[22] Tillett N.D., Hague T. (1999). Computer-vision-based hoe guidance for cereals—An initial trial. J. Agric. Eng. Res..

[23] Åstrand B., Baerveldt A.-J. (2005). A vision based row-following system for agricultural field machinery. Mechatronics.

[24] Benson E.R., Reid J.F., Zhang Q. (2003). Machine vision-based guidance system for agricultural grain harvesters using cut-edge detection. Biosyst. Eng..

[25] Gottschalk R., Burgos-Artizzu X.P., Ribeiro A., Pajares G. (2010). Real-time image processing for the guidance of a small agricultural field inspection vehicle. Int. J. Intel. Syst. Technol. Appl..

[26] Kise M., Zhang Q., Rovira Más F. (2005). A stereovision-based crop row detection method for tractor-automated guidance. Biosyst. Eng..

[27] Sainz-Costa N., Ribeiro A., Burgos-Artizzu X.P., Guijarro M., Pajares G. (2011). Mapping wide row crops with video sequences acquired from a tractor moving at treatment speed. Sensors.

[28] Søgaard H.T., Olsen H.J. (2003). Determination of crop rows by image analysis without segmentation. Comput. Electron. Agric..

[29] Hague T., Tillett N.D., Wheeler H. (2006). Automated crop and weed monitoring in widely spaced cereals. Precis. Agric..

[30] Hough P.V. (1962). Method and Means for Recognizing Complex Patterns. US Patent.

[31] Marchant J.A. (1996). Tracking of row structure in three crops using image analysis. Comput. Electron. Agric..

[32] Hague T., Marchant J.A., Tillett D. (1997). A system for plant scale husbandry. Precis. Agric..

[33] Leemans V., Destain M.-F. (2006). Application of the Hough transform for seed row localisation using machine vision. Biosyst. Eng..

[34] Tellaeche A., Burgos-Artizzu X.P., Pajares G., Ribeiro A. (2008). A vision-based method for weeds identification through the Bayesian decision theory. Pattern Recognit..

[35] Tellaeche A., BurgosArtizzu X.P., Pajares G., Ribeiro A., Fernández-Quintanilla C. (2008). A new vision-based approach to differential spraying in precision agriculture. Comput. Electron. Agric..

[36] Tellaeche A., Pajares G., Burgos-Artizzu X.P., Ribeiro A. (2011). A computer vision approach for weeds identification through Support Vector Machines. Appl. Soft Comput..

[37] Kise M., Zhang Q. (2008). Development of a stereovision sensing system for 3D crop row structure mapping and tractor guidance. Biosyst. Eng..

[38] Vioix J.-B., Douzals J.-P., Truchetet F., Assémat L., Guillemin J.-P. (2002). Spatial and spectral methods for weed detection and localization. EURASIP J. Appl. Sign. Process..

[39] Bossu J., Gée C., Guillemin J.P., Truchetet F. Development of methods based on double Hough transform and Gabor filtering to discriminate crop and weeds in agronomic images. Proceedings of the SPIE 18th Annual Symposium Electronic Imaging Science and Technology.

[40] Bossu J., Gée C., Jones G., Truchetet F. (2009). Wavelet transform to discriminate between crop and weed in perspective agronomic images. Comput. Electron. Agric..

[41] Romeo J., Pajares G., Montalvo M., Guerrero J.M., Guijarro M., Ribeiro A. (2012). Crop row detection in maize fields inspired on the human visual perception. Sci. World J..

[42] Conesa-Muñoz J., Gonzalez-de-Soto M., Gonzalez-de-Santos P., Ribeiro A. (2015). Distributed multi-level supervision to effectively monitor the operations of a fleet of autonomous vehicles in agricultural tasks. Sensors.

[43] Bochtis D.D., Sørensen C.G. (2009). The vehicle routing problem in field logistics part I. Biosyst. Eng..

[44] Conesa-Munoz J., Bengochea-Guevara J.M., Andujar D., Ribeiro A. Efficient Distribution of a Fleet of Heterogeneous Vehicles in Agriculture: A Practical Approach to Multi-path Planning. Proceedings of the 2015 IEEE International Conference on Autonomous Robot Systems and Competitions (ICARSC).

[45] Meyer G.E., Neto J.C. (2008). Verification of color vegetation indices for automated crop imaging applications. Comput. Electron. Agric..

[46] Woebbecke D.M., Meyer G.E., Von Bargen K., Mortensen D.A. (1995). Color indices for weed identification under various soil, residue, and lighting conditions. Trans. ASAE.

[47] Andreasen C., Rudemo M., Sevestre S. (1997). Assessment of weed density at an early stage by use of image processing. Weed Res..

[48] Perez A.J., Lopez F., Benlloch J.V., Christensen S. (2000). Colour and shape analysis techniques for weed detection in cereal fields. Comput. Electron. Agric..

[49] Aitkenhead M.J., Dalgetty I.A., Mullins C.E., McDonald A.J.S., Strachan N.J.C. (2003). Weed and crop discrimination using image analysis and artificial intelligence methods. Comput. Electron. Agric..

[50] Yang C.-C., Prasher S.O., Landry J.-A., Ramaswamy H.S. (2003). Development of an image processing system and a fuzzy algorithm for site-specific herbicide applications. Precis. Agric..

[51] Ribeiro A., Fernández-Quintanilla C., Barroso J., García-Alegre M.C., Stafford J.V. Development of an image analysis system for estimation of weed pressure. Proceedings of 5th European Conference on Precision Agriculture.

[52] Van Evert F.K., Van Der Heijden G.W., Lotz L.A., Polder G., Lamaker A., De Jong A., Kuyper M.C., Groendijk E.J., Neeteson J.J., Van der Zalm T. (2006). A Mobile Field Robot with Vision-Based Detection of Volunteer Potato Plants in a Corn Crop. Weed Technol..

[53] Burgos-Artizzu X.P., Ribeiro A., Tellaeche A., Pajares G., Fernández-Quintanilla C. (2010). Analysis of natural images processing for the extraction of agricultural elements. Image Vision Comput..

[54] Bengochea-Guevara J.M., Burgos Artizzu X.P., Ribeiro A. Real-time image processing for crop/weed discrimination in wide-row crops. Proceedings of RHEA.

[55] Sheikholeslam S., Desoer C. Design of decentralized adaptive controllers for a class of interconnected nonlinear dynamical systems. Proceedings of the 31st IEEE Conference on Decision and Control.

[56] Rossetter E.J., Gerdes J.C. Performance guarantees for hazard based lateral vehicle control. Proceedings of the ASME 2002 International Mechanical Engineering Congress and Exposition.

[57] Pomerleau D.A. (1989). Alvinn: An Autonomous Land Vehicle in a Neural Network.

[58] Sugeno M. (1999). On stability of fuzzy systems expressed by fuzzy rules with singleton consequents. IEEE Trans. Fuzzy Syst..

[59] Zadeh L.A. (1965). Fuzzy sets. Inf. Control.

[60] Fraichard T., Garnier P. (2001). Fuzzy control to drive car-like vehicles. Rob. Autom. Syst..

[61] Naranjo J.E., Sotelo M., Gonzalez C., Garcia R., Sotelo M.A. (2007). Using fuzzy logic in automated vehicle control. IEEE Intell. Syst..

[62] Pradhan S.K., Parhi D.R., Panda A.K. (2009). Fuzzy logic techniques for navigation of several mobile robots. Appl. Soft Comput..

[63] Kodagoda K.R.S., Wijesoma W.S., Teoh E.K. (2002). Fuzzy speed and steering control of an AGV. IEEE Trans. Control Syst. Technol..

[64] Antonelli G., Chiaverini S., Fusco G. (2007). A fuzzy-logic-based approach for mobile robot path tracking. IEEE Trans. Fuzzy Syst..

[65] Digital.CSIC. http://digital.csic.es/handle/10261/110162.

